# Fibroblast Growth Factor Receptor Functions in Glioblastoma

**DOI:** 10.3390/cells8070715

**Published:** 2019-07-13

**Authors:** Ana Jimenez-Pascual, Florian A. Siebzehnrubl

**Affiliations:** European Cancer Stem Cell Research Institute, Cardiff University School of Biosciences, Cardiff CF24 4HQ, UK

**Keywords:** FGFR, review, malignant glioma, brain cancer, astrocytoma, fibroblast growth factor

## Abstract

Glioblastoma is the most lethal brain cancer in adults, with no known cure. This cancer is characterized by a pronounced genetic heterogeneity, but aberrant activation of receptor tyrosine kinase signaling is among the most frequent molecular alterations in glioblastoma. Somatic mutations of fibroblast growth factor receptors (*FGFRs*) are rare in these cancers, but many studies have documented that signaling through FGFRs impacts glioblastoma progression and patient survival. Small-molecule inhibitors of FGFR tyrosine kinases are currently being trialed, underlining the therapeutic potential of blocking this signaling pathway. Nevertheless, a comprehensive overview of the state of the art of the literature on FGFRs in glioblastoma is lacking. Here, we review the evidence for the biological functions of FGFRs in glioblastoma, as well as pharmacological approaches to targeting these receptors.

## 1. Introduction

Fibroblast growth factors (FGFs) were first isolated from bovine brain extracts in 1939 and characterized by their ability to induce proliferation of fibroblasts [1]. It took another 50 years to discover and clone the first of their cognate receptors [2]. FGFRs control many biological functions, including cell proliferation, survival, and cytoskeletal regulation (for review, see [3]). FGFR signaling is important during embryonal development of the CNS, and as a survival mechanism for adult neurons and astrocytes [4,5,6]. Furthermore, FGFR signaling was found to promote self-renewal and fate specification of neural stem cells [7].

In many cancers, *FGFR* aberrations have been implicated in tumor development and progression [8,9], and include *FGFR* overexpression, amplification, mutations, splicing isoform variations, and *FGFR* translocations [10,11]. While *FGFR* genomic alterations have been identified in many solid tissue cancers, such events remain rare in glioblastoma (GBM) (Table 1) [12]. Nonetheless, FGFR expression changes in astrocytes can lead to malignant transformation and GBM progression due to the activation of mitogenic, migratory, and antiapoptotic responses [13,14,15]. Of note, fusions between *FGFR* and *TACC* (transforming acidic coiled-coil containing proteins) genes were shown to be oncogenic in GBM [16], and occur in about 3% of GBM patients [12]. Whole-genome analyses of patient samples have revealed that the number of *FGFR* mutations and amplifications are generally very low in GBM (*FGFR1*: 51/3068 samples, *FGFR2*: 12/2662; *FGFR3*: 16/2887; *FGFR4*: 9/2456; cancer.sanger.ac.uk; [17]). Not only are oncogenic mutations in *FGFRs* rare in GBM, the lack of *FGFR* passenger mutations (i.e., mutations not providing a survival benefit) suggests that these are selected against, and that the maintenance of dynamic FGFR signaling is important for the development and/or progression of GBM.

We hypothesize that the neurodevelopmental and cell survival functions of this signaling pathway are at least partly conserved in GBM. Thus, dynamic FGFR signaling needs to be maintained for the survival of GBM cells, and therefore evolutionary pressure selects against both activating and inactivating mutations. Here, we review the current literature on FGFRs in GBM, and the evidence for differential functions of individual FGFRs in brain tumor progression.

## 2. FGFR Structure

**Figure 1:**
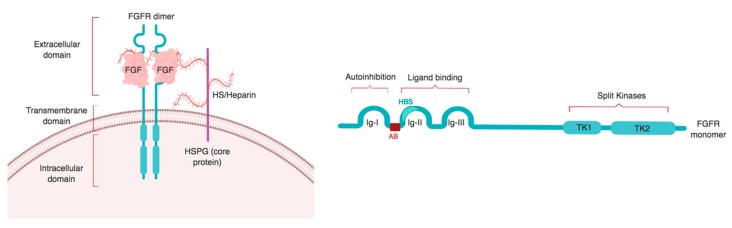
Domain structure of FGFRs: an extracellular domain containing ligand binding site is followed by a single transmembrane domain, and an intracellular domain containing split tyrosine kinases. Left panel: organization of the FGF–FGFR complex at the cell surface. The FGF–FGFR complex is stabilized by a heparin/HS chain of the HS proteoglycan (HSPG). Right panel: The extracellular domain of the receptor is composed of three Ig-like domains: Ig-I, Ig-II, and Ig-III. Ig-I has autoinhibitory capacity while Ig-II and Ig-III form the ligand binding domain. Ig-II contains the heparin/HS binding site (HBS) and is separated from Ig-I by an acid box (AB). The cytoplasmic domain is formed by two tyrosine kinases: tyrosine kinase 1 (TK1) and tyrosine kinase 2 (TK2). Image created with biorender.com.

There are four known FGFRs, FGFR1–4, which are membrane-bound receptor tyrosine kinases (RTKs). A fifth member of the FGFR family, FGFRL1, is lacking a transmembrane domain and is therefore soluble. FGFRL1 acts as an antagonist to FGFR signaling [19,20]. Structurally, FGFR1–4 consist of three different domains: an extracellular ligand binding domain, a transmembrane domain, and an intracellular domain that interacts with cytoplasmic molecules and transduces FGFR signaling [8,21,22] (Figure 1).

The extracellular domain can bind FGF ligands, heparan sulfate (HS), and extracellular matrix molecules, which can act as a scaffold to enable receptor binding of specific FGFs. It is divided into three immunoglobulin-like (Ig) loops: Ig-I, Ig-II, and Ig-III (also called D1, D2, and D3) [23]. Ig-I is linked to Ig-II by a stretch of 30 acidic residues called the acid box, a unique region of FGFRs [10,24]. Ig-I and the acid box have receptor auto-inhibitory functions [25,26,27] while the Ig-II and Ig-III subdomains form the ligand binding site of the receptor [3,15,18,28,29]. Ig-II contains the heparin/HS binding region and FGF binding activity site, while the junction between Ig-II and Ig-III controls heparin and FGF affinity [21,30,31,32,33] (Figure 1).

Multiple FGFR isoforms are generated by alternative splicing of the region encoding for the extracellular domain. This modifies the affinity and sensitivity of the receptors for different FGF ligands [34,35]. Thus, an array of FGFR isoforms is created that can fine-tune the response of cells to the large number of potential FGF ligands available in their specific environment. FGF sensitivity is further modified by co-receptors, such as Klotho family members, which are required for binding of endocrine FGFs [36,37,38].

Alternatively-spliced β isoforms of FGFR1 or FGFR2 are produced by the exclusion of the Ig-I domain, which is encoded by exon 3. Due to the auto-inhibitory function of the Ig-I domain, the β isoform has considerably higher affinity for FGFs, and is oncogenic [39,40]. Retention of the Ig-I domain creates FGFR α isoforms [28].

**Figure 2:**
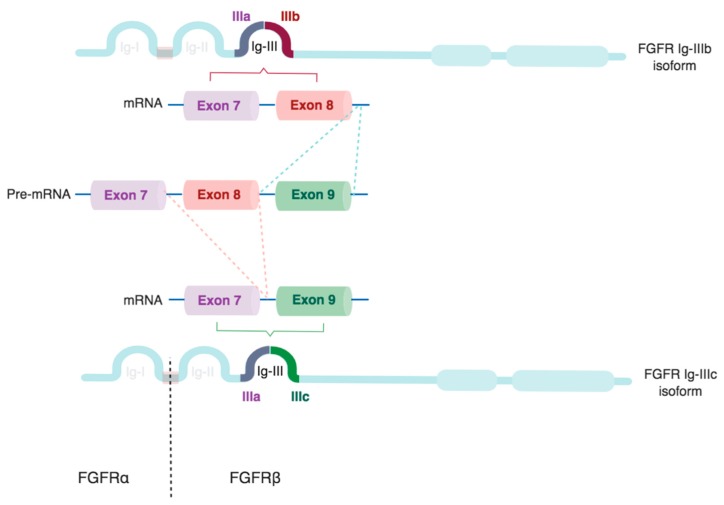
Schematic representation of FGFR splice isoforms. The Ig-III domain of FGFR1–3 is encoded by exons 7–9. Exon 7 encodes Ig-IIIa, which consists of the N-terminal half of the Ig-III loop. The C-terminal half is formed by the IIIb or IIIc sequence, which is generated by the selective inclusion of exons 8 or 9, respectively. Truncation of the Ig-I loop creates FGFRβ isoforms (dotted line), while the full-length receptor is termed FGFRα. Image created with biorender.com.

Additionally, alternative splicing generates two isoforms of the Ig-III domain, known as Ig-IIIb and Ig-IIIc, in FGFR1–3, but not FGFR4 [10,41,42,43]. Ig-IIIb and Ig-IIIc are generated by exon skipping, and are encoded by exons 8 and 9, respectively (Figure 2). By contrast, exon 7, encoding Ig-IIIa, is present in all splice variants. Different splice-regulatory proteins have been identified that control the splicing of Ig-IIIb, such as regulatory RNA-binding protein (RBP), and the epithelial splicing regulatory proteins (ESRP1/2) [32,40,44].

The expression of FGFR splice variants is tissue-dependent. For example, the Ig-IIIb isoform is more prevalent in epithelial tissues, while Ig-IIIc is preferentially expressed in mesenchymal ones [22,24,32]. Switching of epithelial and mesenchymal isoforms occurs during epithelial–mesenchymal transition, which is known as the IIIb/IIIc switch. Hence, FGFR isoform expression is also related to tissue plasticity, and changes during tissue growth, proliferation, and remodeling [45].

The FGFR transmembrane domain is crucial for transferring the signal from the extracellular to the intracellular domain, the latter consisting of a juxtamembrane domain, two tyrosine kinase domains, and the C-terminal tail [10,32,46,47]. FGFR domains are highly conserved among receptors, and the tyrosine kinase domain shares the highest homology. The Ig-III domain is also highly conserved, especially between FGFR1 and FGFR2 [48].

The binding of FGF ligands to HSPGs causes FGFR dimerization and activation in the -COOH receptor tail of the cytoplasmic tyrosine residues by phosphorylation [49,50]. For instance, autophosphorylation of FGFR1 tyrosine (Y) residues occurs in three steps. Firstly, phosphorylation of Y653 leads to a 50–100-fold increase of the catalytic core activation of the intracellular domain. Secondly, Y583, Y463, Y766, and Y585 sites are consequently phosphorylated, and finally, the second tyrosine kinase domain phosphorylation increases the tyrosine kinase activity 10-fold. This sequence of autophosphorylation follows a specific and controlled order that, if deregulated, can induce malfunction of the pathway [49].

Different models have been proposed for FGFR dimerization depending on the ligand–heparin–receptor complex, specificity between the ligand and the receptor, and the heparin length required for the binding [21,24]. In the first model, HS increases the association between the receptor and the high affinity binding site of the ligand, forming a ternary complex (1:1:1 FGF–HS–FGFR) that then interacts with a second receptor, inducing FGFR dimerization through the FGF low affinity binding site (2:1:1 FGFR–HS–FGF). On the other hand, the symmetrical model suggests that two individual ternary complexes are formed. The FGFR dimerization will then occur by FGFR–FGFR direct interaction, FGF ligand interaction, or by HS–HS link (2:2:2 FGFR–HS–FGF). In this model, HS enhances FGFR dimerization, but it is not crucial. Finally, according to the asymmetric model, HS attaches to two FGF–FGFR complexes, binding both FGFs but only one of the receptors (2:1:2 FGFR–HS–FGF) [21]. Therefore, although different models have been suggested, more research is needed to clarify the stoichiometry of FGFR dimerization [21,24,51].

## 3. FGFR Signaling Cascade

FGF–FGFR stimulates cell signaling pathways related to cell proliferation, survival, cytoskeletal regulation, and FGFR degradation [10]. Cell proliferation is mainly induced by RAC/JNK and RAS–MAPK signaling pathways [3]. RAC kinases can be activated by the transient phosphorylation of CRK, which simultaneously stimulates RAC phosphorylation trough DOCK1 or SOS/RAS [10]. RAC kinases promote proliferation by the activation of JNK and p38. Alternatively, the RAS/RAF/MEK/ERK signaling pathway can be activated by the FRS2–GRB2–SOS–SHP2 complex assembly or byPKC activation through PLC phosphorylation [18,22].

**Figure 3:**
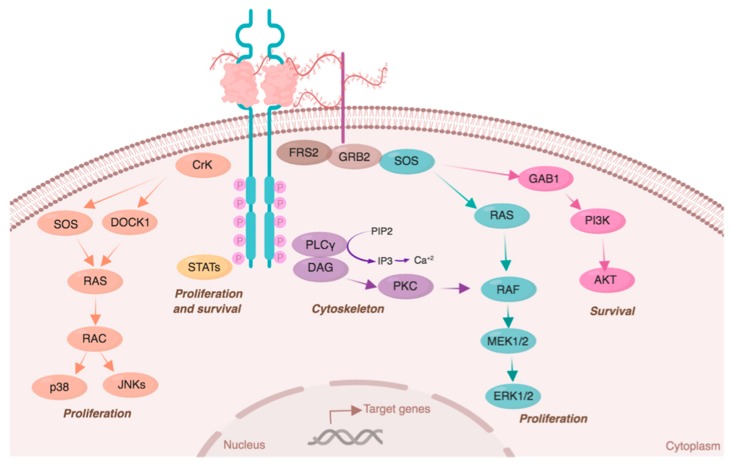
FGFR signaling pathway. After ligand binding, FGFRs dimerize and activate multiple signal transduction pathways. Each pathway induces the expression of specific target genes related to cell proliferation (STATs, RAS/p38/JNKs, and RAS/MAPK/ERK), survival (STATs and PI3K/AKT), and cytoskeleton regulation (PLC/Ca^2+^). Kinases are color-coded according to their specific signaling pathway. Image created with biorender.com.

Cell survival is mainly promoted by phosphorylation of PI3K/AKT signaling through the FRS2–GRB2–GAB1 complex. Finally, FGFRs are also implicated in cytoskeletal regulation, as PLC phosphorylation leads to the hydrolysis of PIP_2_ into IP3, inducing calcium release [18] (Figure 3).

**Figure 4:**
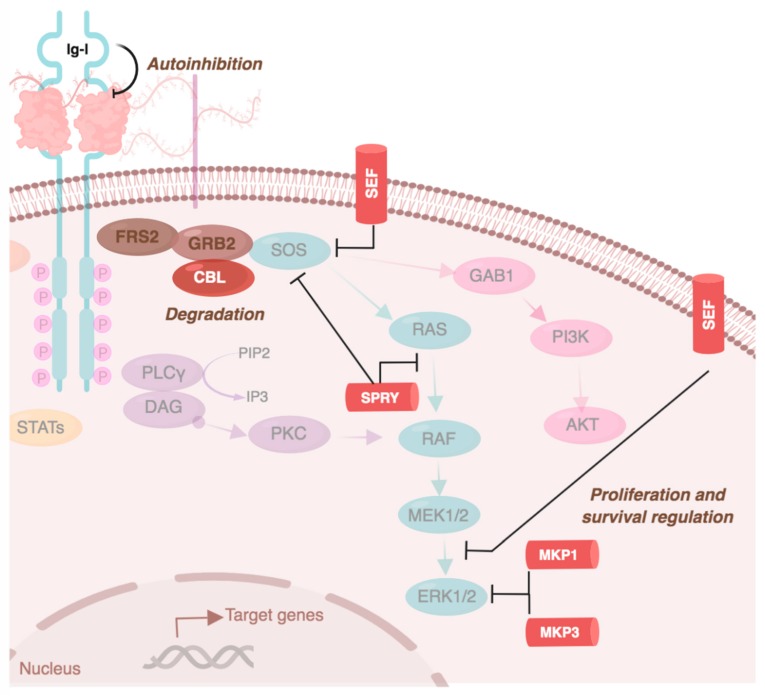
FGFR signaling pathway regulation. FGFR signaling is negatively regulated, partly by CBL (inducing FGFR degradation after receptor internalization), by SEF, SPRY, MKP1, and MPK3 (which negatively regulate proliferation and survival related pathways). FGFRs can also regulate their own activation due to the autoinhibitory function of Ig-I. Image created with biorender.com.

Because FGFR signaling acts upon many biological functions, a regulatory system that controls its timing, spread, and balances its activation is required. This is important as the activation of the signaling cascade depends on FGFR expression and localization to the cell membrane. Therefore, receptor availability depends on the balance between its recycling and degradation rate, which differ among receptors. One of these regulatory systems is FGFR internalization or constitutive endocytosis. FGFR synthesis occurs at a higher level than its internalization. However, after ligand-binding, FGFR internalization from the plasma membrane accelerates [52]. FGFR internalization is primarily mediated by clathrin-dependent endocytosis and requires the SRC–FRS2 complex [53]. The internalization rate depends on the receptor type—FGFR1 has the highest internalization rate and FGFR3 the lowest. Endocytosis of activated FGFRs involves detachment from the SRC complex [54]. FGFRs can then re-translocate to the cytosol, mitochondria, nucleus (to directly regulate gene expression), or to the endosomal compartment for receptor degradation [52]. The latter requires interaction between the FRS2–GRB complex and CBL, and is receptor-independent [22] (Figure 4). Indeed, FGFR1 has more ubiquitination sites than FGFR4, so its degradation rate is likely higher [10].

Other regulatory systems of FGFR signaling are the negative regulators SEF, SPRY1/SPRY4, and MKP1/MKP3. The activation of cell proliferation is counterbalanced by SEF, which negatively regulates ERK and AKT activation [18]. Similarly, SPRY1/SPRY4 reduce proliferation by directly interacting with RAS/RAF kinases or by blocking the FRS2–GRB2–SOS–SHP2 complex. MKP1 and MKP3 also attenuate FGFR signaling by dephosphorylating MAPK and ERK [10] (Figure 4).

FGF signaling is also negatively regulated by the autoinhibitory (Ig-I) domain of the receptors. This is controlled by the electrostatic interactions between the negatively charged acid box with the highly basic heparin binding site in Ig-II [25]. This complex blocks the heparin–FGF binding, minimizing FGFR activation. The auto-inhibitory capacity is crucial for the modulation of the pathway, as the high amount of HSPGs from the cell surface and the extracellular matrix increases the probabilities of FGF–heparin binding and activation of the RTK cascade [24].

Other factors involved in FGFR pathway regulation are the ligand affinity for the receptor and the ligand amount and availability. Extracellular FGFs are protected and stored by HS proteoglycans. Heparanases are directly involved in FGF signaling regulation, as they cleave the HS chain, thus releasing FGFs in the vicinity of cells. Depending on the cell type and the growth factor released, heparanases are therefore involved in cell growth, differentiation, or stemness maintenance [55]. Likewise, sufficient amounts of ligand and heparin/HSPGs are necessary for stabilizing FGFR dimerization. The necessary ligand concentration is dependent on the ligand-binding affinity of the FGFRs, which depends on the FGFR splice isoforms [3,8,42]. For example, FGF2 activates both FGFR1 IIIb and IIIc isoforms, while it has a higher affinity for the isoform IIIc in FGFR2 and FGFR3 [42].

## 4. Crosstalk between FGFRs and Other Cell Surface Molecules

FGFRs can be modulated independently of their ligands by integral cell membrane proteins, such as G-protein-coupled receptors (GPCRs), cell adhesion molecules (CAMs), and other RTKs, which play a crucial role in the induction of specific cell responses and fate during development and cancer [56]. GPCRs can transactivate FGFRs by promoting the activation of matrix metalloproteinases, resulting in cleavage of FGFs, or by directly interacting with FGFRs [57]. GPCR-mediated FGFR1 transactivation is associated with neuronal differentiation, neurite growth, and synaptic plasticity [56]. FGFR1 modulation by GPCRs (e.g., CB1A, 5-HT1A, and mAChR) involves the activation of the SRC-ERK1/2 pathway [57,58,59]. In C6 glioma cells, crosstalk between the mu-Opioid receptor and FGFR1 was shown to activate this signaling cascade, but the specific mechanism is not yet completely understood [60].

FGFR activity is also modulated by CAMs, cell surface proteins that regulate cell–cell interactions and motility. Importantly, the FGFR acid box region is required for the CAM/FGFR interaction [61], hence the FGFR β isoforms cannot be transactivated by CAMs. CAMs of the integrin, cadherin, and immunoglobulin (e.g., NCAM and L1-CAM) superfamilies signal through FGFRs to induce neurite outgrowth, cell survival, and oncogenesis [61,62,63,64,65,66]. Integrins signaling can activate cell proliferation, survival, and invasion [67], and integrin α6 [68] and α7 [69] have been linked to GBM cancer stem cells (GSCs). A recent study suggested that integrin α6 regulates the expression of FGFR1 through ZEB1 and YAP transcription factors [70]. N-cadherin stabilizes FGFR1 and decreases its internalization, thus promoting invasion in breast cancer cells, and N-Cadherin/FGFR crosstalk promotes neurite outgrowth [71]. Of note, the stem cell transcription factor ZEB1 regulates N-Cadherin expression, which is associated with EMT and invasion. It is tempting to speculate that N-Cadherin/FGFR1 interactions could constitute a positive feedback loop in GSCs through the activation of ZEB1 and subsequent induction of N-Cadherin and FGFR1 expression [70,72] (see also Section 5.1). NCAMs physically associate with FGFRs and inhibit the high-affinity binding between these receptors and their canonical ligands [56,73]. Furthermore, polysialic acid-NCAM (PSA-NCAM) has been described as a marker of GBM patient prognosis [74]. This study showed that a targeted expression of PSA-NCAM in C6 glioma cells resulted in increased levels of Olig2, a transcription factor associated with GSCs [75]. While it remains unclear whether this was the result of FGFR transactivation by PSA-NCAM, we have recently shown that OLIG2 can be induced by FGFR1 signaling [72]. Furthermore, the L1-CAM/FGFR1/Anosmin-1 complex regulates neurite branching [76,77,78] and L1-CAM-mediated FGFR1 transactivation induces glioma cell proliferation and motility [79].

Crosstalk between FGFRs and other RTKs, such as EPHs and PDGFRs, has been identified in Y2H screens and endothelial cells [80,81]. FGFR/RTKs form a heterocomplex in which the tyrosine kinase domain of the FGFR is phosphorylated by the other receptor [56]. EPHA4 transactivated FGFR1 in the U251 glioma cell line, promoting cell growth and migration, and EPHA4 expression is increased in glioma [82]. Less is known about potential crosstalk between other RTKs and FGFRs in GBM. In summary, FGFR activity can be modulated non-canonically by other cell surface proteins, resulting in the activation of intracellular signaling pathways and cell responses associated with FGFR signaling.

## 5. Expression and Functions of FGFRs in Glioblastoma

Gene expression analysis of TCGA data (GBM 540) revealed profound heterogeneity of *FGFR1–4* expression across GBM patients [72]. Below, we discuss the evidence for the functions of individual FGFRs in glioma.

### 5.1. FGFR1

Yamaguchi et al. found that expression of FGFR1 increases with WHO grade in astrocytomas [39], and increased FGFR1 levels in GBM are not due to amplification of the *FGFR1* gene [83].

In addition to the increased expression of FGFR1 in malignant gliomas, the ratio of alternatively spliced FGFR1 α/β isoforms changes with progression to more aggressive brain cancers. While FGFR1 α is the predominant isoform in normal brain and low-grade gliomas, high-grade gliomas show a shift towards the expression of FGFR1 β [13,39]. Loss of the FGFR1 α exon increases the receptor–ligand affinity [28], thus, changes in alternative splicing may contribute to GBM malignancy by increasing the sensitivity of tumor cells to FGFs present in their environment.

Functionally, FGFR1 expression in malignant glioma has been associated with increased migration of cancer cells [82]. In this study, a high expression of EPHA4 in glioma cells was found to potentiate FGF2–FGFR1 signaling and promoted cell growth and migration through the AKT/MAPK and RAC1/CDC42 pathways, respectively. Data from our lab support that FGFR1 loss results in reduced tumor invasion in vivo (Jimenez-Pascual and Siebzehnrubl, unpublished observation).

Loilome and colleagues identified FGFR1 as a potential transducer of FGF2 effects on glioma cell proliferation [84], but whether other FGFRs also contribute was not directly tested. Nevertheless, the pharmacological inhibition of FGFR signaling significantly reduced tumor cell growth in a range of established and patient-derived glioma lines.

The malignancy-promoting effects of FGFR1 were further demonstrated in a study that found FGFR1 signaling promoting radioresistance in glioma cell lines through PLC1γ and HIF1α [85]. FGFR1 expression is regulated by the stem-cell associated transcription factor ZEB1 [70], suggesting that FGFR1 may be associated with GBM cancer stem cells. We recently performed a comprehensive analysis of the functions of FGFR1-3 in GBM and found that FGFR1 indeed is preferentially expressed on GSCs, where it regulates the expression of the critical stem cell transcription factors SOX2, OLIG2, and ZEB1, thereby promoting tumorigenicity in vivo [72]. In summary, FGFR1 is a key regulator of tumor growth, invasion, therapy resistance, and cancer stemness in malignant glioma.

### 5.2. FGFR2

While FGFR1 is mainly expressed on neurons [6], FGFR2 is the primary FGFR on astrocytes [5]. In contrast to FGFR1, FGFR2 expression decreases with glioma grade [43]. Reduced expression of FGFR2, as well as its IIIb and IIIc isoforms, is associated with a higher tumor grade and poorer survival in glioma patients [43]. Tumors with a higher expression of FGFR2 showed significantly less proliferation, as identified by Ki-67 staining, but whether there is a direct link between FGFR2 signaling and slowing or exiting the cell cycle remains unclear. By contrast, experimental tumors derived from in vivo implantation of C6 glioma cells exhibited decreased tumor growth after inhibition of FGFR2 signaling by a dominant negative construct [86].

Our recent analysis of cell-surface FGFR expression patterns in GBM stem cell lines indicates that FGFR2 is nevertheless highly prevalent on GBM cells in vitro [72], but it remains to be tested whether FGFR2 loss results in increased proliferation and/or tumorigenicity. Loss of FGFR2 is associated with a loss of Chr. 10q, which in and of itself carries an unfavorable prognosis [87]. It is therefore conceivable that FGFR2 loss is not causally linked to reduced patient survival, and further work is needed to clarify the functional relevance of FGFR2 signaling in GBM.

### 5.3. FGFR3

In a small subset of GBM patients, fusion of the *FGFR3* and *TACC3* genes generates an oncogenic *FGFR3* form [16]. In rare cases, fusion between *FGFR1* and *TACC1* can occur as well [88]. In *FGFR3–TACC3*, the FGFR tyrosine kinase domain is fused to the TACC coiled-coil domain, resulting in constitutive activation of the fused receptor. Small-molecule FGFR inhibitors were effective at blocking tumor growth where *FGFR–TACC* fusion occurred, indicating that the fused receptor is causally linked to tumor development. Overall, *FGFR–TACC* fusions are found in ~3% of gliomas and are mutually exclusive with *EGFR* amplifications. Recently, it has been shown that *FGFR–TACC* fusions affect cell metabolism, activating oxidative phosphorylation and mitochondrial activity [89]. Of note, we have recently found that GSCs preferentially utilize oxidative phosphorylation and mitochondrial respiration [90], and therefore it would be interesting to investigate whether *FGFR–TACC* fusions also affect stemness pathways in GBM.

Whether FGFR3 has specific functions that differ from FGFR1 or -2 in GBM, and/or whether signaling through FGFR3 activates specific downstream signaling pathways remains unclear. Of note, global transcriptomic analysis of TCGA and CGGA datasets found increased expression of *FGFR3* in the classical and neural subtypes of GBM [91]. Gene ontology analysis showed an association of FGFR3 expression with biological processes of cell differentiation in this study.

A recent study investigating gene expression using single-cell RNA-Seq in GBM patients found that *FGFR3* expression is five-fold higher in invasive GBM cells compared to the tumor core [92]. Indeed, *FGFR3* was the second highest differentially expressed gene between invasive and tumor core GBM cells. While this suggests that FGFR3 may be functionally associated with tumor invasion, whether FGFR3 signaling is driving GBM invasion remains to be shown.

### 5.4. FGFR4

Very little evidence exists of the expression of FGFR4 in GBM. An early study found increased expression of the FGFR4 protein, but not mRNA, with increasing grade in astrocytoma [93]. Another study demonstrated the expression of FGFR4 across different GBM cell lines [84]. We recently investigated FGFR protein expression in GBM cells [72]. In our study, FGFR4 was not detectable by western blot in primary patient-derived GBM cells, and analysis of GBM patient data in the TCGA dataset showed heterogeneous, but overall low expression of *FGFR4*. Moreover, we could not find differences in survival when stratifying patients for *FGFR4* high or low expression. More research is needed to fully characterize whether FGFR4 is expressed on subsets of GBM cells, and whether it is functional in these cancers.

## 6. FGFRs as Therapeutic Targets in GBM

Oncogenic FGFR signaling promotes malignancy in many cancers, including CNS malignancies. Thus, pharmacological targeting of FGFRs may be therapeutically beneficial. A number of RTK inhibitors have been developed that show selectivity of FGFRs over other RTKs [18]. While some small-molecule inhibitors also target non-FGFR RTKs (e.g., dovitinib, levatinib, brivanib), others are selective for FGFR1–3 (e.g., PD173074, BGJ398, AZ4547, JNJ-493). To date, no small-molecule inhibitors exist with good selectivity for individual FGFR subtypes or isoforms [18].

Recently, a study identified FGFR signaling as a potential therapeutic target in pediatric glioma using a large-scale shRNA screen [94]. In this study, FGFR inhibitors (AZ4547, dovatinib, PD173074, ponatinib) were more effective in reducing the growth of pediatric glioma cells in vitro than the first-line chemotherapeutic agent Temozolomide.

The selective FGFR inhibitors AZ4547 and BGJ398 have been tested in clinical phase I/II (NCT028224133) and phase II trials (NCT01975701), respectively. AZ4547 was trialed in patients with recurrent IDH wild-type gliomas with *FGFR1–TACC1* or *FGFR3–TACC3* fusions, but this trial was suspended after analysis of the data from the first 12 patients. A trial of BGJ398 in malignant glioma patients with *FGFR1–TACC1* or *FGFR3–TACC3* fusion, and/or activating mutation in *FGFR1*, -*2*, or -*3*, was completed, but so far, no results have been published.

A phase I/II trial of the irreversible FGFR inhibitor TAS-120 (NCT02052778) is currently recruiting patients with advanced solid tumors, including brain tumors. As with the AZ4547 and BGJ398 trials, the focus of this trial is on patients with *FGFR* gene fusions or activating mutations.

Due to the prevalence of FGFRs on many CNS cells, and the importance of FGFR signaling for CNS cell survival, as well as the apparent intratumoral and intertumoral heterogeneity of FGFR expression in GBM, it will be interesting to see whether FGFR inhibitors are successful as monotherapy. Based on the evidence implicating FGFR1 as a GSC regulator, it is further tempting to speculate whether the targeted inhibition of FGFR1 in combination with conventional chemo/radiotherapy could prevent or delay recurrence in GBM.

## 7. Conclusions

Gene expression profiling and whole-genome sequencing data indicate that all four FGFRs are expressed to varying degrees in GBM, underlining the heterogeneity of this disease. Several studies have documented that high-grade gliomas show an increased expression of *FGFR1*, and decreased expression of *FGFR2* [13,39,43,72,83], but in almost all cases, *FGFR* expression was detected at a global level, and limited or no effort was made to further identify *FGFR* splice isoforms. A recent report investigating gene expression at the single-cell level [92] found that *FGFR3* was expressed the second-highest in invasive GBM cells. This illustrates that much more research is needed to unravel the functions of individual FGFRs and their splice isoforms in brain cancers in general, and GBM in particular.

To what extent signaling through individual FGFRs contributes to disease progression, and whether individual FGFRs and/or isoforms activate specific pathways linked to different pathobiological aspects of these cancers (e.g., invasion, tumor initiation, therapy resistance) remains largely unknown. Yet, the fact that FGFR subtypes are differentially expressed on cellular subpopulations within the same tumor suggests that individual FGFRs may have divergent functions in GBM [72,92].

The strongest evidence by far indicates that FGFR1 is an important contributor to poor outcome in GBM, and FGFR1 signaling is linked to cancer stemness, invasion, and radioresistance [70,72,85]. However, recent evidence from TCGA datasets highlights that *FGFR1–4* are expressed to varying degrees and in different combinations in patient samples [72]. This calls for a more detailed analysis of FGFR distribution across GBM patients and within individual tumors. It is conceivable that different combinations of FGFR subtypes and splice isoforms mediate and/or modulate different aspects of FGF signaling in GBM cells. Only a comprehensive analysis of the cell-surface expression of FGFRs at the single-cell level will dissect the intratumoral heterogeneity of these receptors, and thus provide the foundation for new, targeted approaches to blocking FGFR signaling for glioma therapy.

## Figures and Tables

**Table 1:** Common *FGFR* genomic aberration in solid tumors. FGF signaling deregulation is involved in the development of many different human cancers. Four FGFR genomic alterations are represented in this table: gene amplification, point mutations, chromosomal translocations, and FGFR splicing isoforms. Each FGFR alteration is linked with the most significant cancers that contain those alterations. The role of the majority of the discovered point mutations in FGFR is unknown in cancer. Adapted from [10,18].

Gene	Gene Amplifications	Point Mutations	Chromosomal Translocations	Splice Variants
*FGFR1*	Breast, ovarian, bladder, and lung cancer	Majority of cancers. Example: Melanoma	Stem cell leukemia/lymphoma (SCLL), GBM	IIIc: small cell lung carcinoma Iβ: breast cancer and GBM
*FGFR2*	Breast, gastric, lung cancer	Majority of cancers. Example: Endometrial carcinoma		IIIb: breast, endometrial, cervical, lung, pancreatic and colorectal cancer IIIc: prostate cancers
*FGFR3*	Bladder cancer	Majority of cancers. Example: bladder cancer	GBM, T-cell lymphoma and bladder	IIIc: bladder cancer
*FGFR4*	Colorectal cancer	Majority of cancers. Example: metastatic breast cancer and rhabdomyosarcoma		
